# Culpability, blame, and stigma after pregnancy loss in Qatar

**DOI:** 10.1186/s12884-019-2354-z

**Published:** 2019-06-26

**Authors:** Nadia Omar, Stella Major, Mona Mohsen, Halima Al Tamimi, Faten El Taher, Susie Kilshaw

**Affiliations:** 1 Medical Research Center, Hamad Medical Corporation, Doha, Qatar; 2 Department of Medical Education, Weill Cornell Medicine-Qatar, Education City, P.O. Box 24144, Doha, Qatar; 3 Interim Translational Research Institute, Hamad Medical Corporation, Doha, Qatar; 4 The Women’s Hospital (Hamad Medical Corporation)-Qatar, Doha, Qatar; 5 University College London-Department of Anthropology, 14 Taviton Street, London, WC1H 0BW United Kingdom

**Keywords:** Blame, Stigma, Guilt, Culpability, Pregnancy loss, Miscarriage, Qatar

## Abstract

**Background:**

Following a miscarriage many women report feeling guilty and culpable for what has happened particularly when aspects of societal blame and stigma are involved. This research investigated the impact of cultural context on the experience of miscarriage. In particular, it focused on how elements of stigma and blame are linked to notions of miscarriage etiology and risk among Qatari women.

**Methods:**

The research used an ethnographic approach. The data was collected over 18 months of fieldwork in Qatar, using semi-structured face to face interviews, and participant observation. A purposive sample of 40 women (primary participants) who had recently miscarried, participated in the study. Potential subjects were initially identified in the Women’s Hospital and were consented, and then interviewed in Arabic either in the hospital or at their preferred location. The interviews were audio recorded, transcribed and translated into English. Additional key interviews were performed with 20 secondary participants related to the miscarriage cohort including family members and husbands. Inductive thematic analysis of content was performed manually to extract themes.

**Results:**

Two main themes emerged from the material looking specifically at miscarriage aftermaths: rhetorics of blame, self-blame and feelings of guilt; and miscarriage attitudes. Overall society is sympathetic and miscarriage is seen as normal and not particularly worrying, but understood to be upsetting to women. However, findings suggest there is some ambivalence around blame, culpability and stigma applied to miscarriage; some participants perceived miscarriage as a relatively normal and common event, whereas, others felt that miscarriage is resounding stigma and shame.

**Conclusion:**

Miscarriage aftermaths are embedded in social, cultural and religious frameworks in relation to notions of risk and causation. Attention should be paid to ensure women and those around them are given appropriate and robust information about miscarriage causation to deflect discourses of blame that may be employed and reduce harm to women who suffer miscarriage.

## Background

At least one in four women with clinically recognized pregnancies experience miscarriage in their lifetimes [1, 2]. Despite its commonality, pregnancy loss in Western societies is often shrouded in silence and ambiguity and rarely discussed in public [3–10]. In addition to its potential physical and emotional harm to the woman and family members [11], many women feel a sense of responsibility or guilt following a miscarriage [7, 8, 12, 13]. It can be associated with loss of self-esteem as women feel that they are unable to fulfill motherhood roles [14, 15]. Miscarriage is a complex experience that takes place in social worlds and thus imbued by social meanings [4, 16–18]. Research shows that societal norms, policies and legislation impact a variety of aspects pertaining to pregnancy loss [9, 16, 18, 19]. Medical models of pregnancy emphasize the ability to control reproduction and leads to assumptions around positive outcomes in pregnancy [20, 21], making pregnant women’s behaviour the target of intense personal and societal scrutiny [22]. Such an emphasis on control and assumptions around pregnancy as success may contribute to a woman’s sense of responsibility for fetal outcome and self- blame in the case of poor outcomes [5, 23].

In Qatar, women’s identities and self-worth are strongly tied to their potential for procreation [4, 16, 20]. As per the recommendations of the 2009 Qatari population policy, fertility is encouraged among Qatari citizens [24]. The total fertility rate in Qatar is the highest in the Arab Gulf States, with it reaching 3.2 in 2015 [25]. Qatari ideas around conception suggest that pregnant women are supposed to remain in a calm state, and should protect their pregnancies; therefore, a good pregnancy is surveyed and cared for by the woman and the medical profession [20]. Given the religious, cultural, and state emphasis on pronatalism^1^ and a women’s role in reproduction, reactions of guilt and blame following a miscarriage are likely.

When dealing with miscarriage, health care professionals rarely consider the integration of physical and social aspects of this experience [8, 27]. Medical anthropologists have shown the way in which health is affected by interpersonal interactions, cultural norms and social institutions, micro and macro politics, and the forces of their wider social networks [28]. Scholarly work that looks at miscarriage in a variety of cultural context (e.g. [29–31]), suggest that experiences of pregnancy loss are culturally contingent, which is supported by more recent work of Van der Sijpt, Kilshaw et al., and Quershi [9, 32, 33]. Therefore, understanding the socio-cultural context and perceptions of adverse pregnancy outcomes is important for informing the best approaches for public health programs. However, the body of work on miscarriage is scant, and those that do exist focus primarily on the Euro-American context, although this is now changing [33–35].

This paper presents a different context than what was discussed in the literature and shows variations to miscarriage experience, but also continuities. It is derived from data on participants’ discussions of stigma and blame in Qatar and considers how this links with notions of miscarriage causality and risk among Qatari women. The ethnographic approach of the study sheds new light on the experience of miscarriage in Qatar. It contributes to a growing literature on women’s health in the Middle East and to the scant literature on reproduction and pregnancy loss. The bigger aim is to better understand Qatari notions of blame and responsibility around miscarriage in order to inform care for women in Qatar and beyond.

## Methods

The project involved over 18 months ethnographic research in Qatar (March 2013 to September 2014). Fieldwork consisted of two phases; the first phase involved 20 pregnant women as it was important to gain information about pregnancy and the notions of the fetus, more generally in order to better understand what is lost when pregnancy fails. The second phase which is the main address of this paper, focused on women who had experienced pregnancy loss. A purposive sample of 40 Qatari women between 18 to 50 years old (primary participants) who had recently (in the past 6 months) experienced a first trimester miscarriage (prior to 20 weeks of gestation), participated in the study. Women with a recent history of three or more consecutive miscarriages were excluded from the study, as these may constitute patients with an underlying medical cause for the miscarriage otherwise referred to as “high risk” women, and this study is looking at understanding miscarriage in the context of “normal risk women”. Also, women with recurrent pregnancy losses are seen as more vulnerable by various ethics bodies, so we decided not to include them. Interviews with key family members (secondary participants) such as husbands (*n* = 7), sisters, mothers and mothers in law (*n* = 13) provided additional material on Qatari experience of loss and helped to better understand the context of miscarriage in the family and its impact on the expectations on womanhood in Qatar. For reasons of cultural sensitivity and privacy, husbands and family members were recruited through the primary participants. The intention was to include as many husbands as possible, however, due to the nature of the Qatari society it was difficult to recruit, either because they or their wives did not want then to participate.

A variety of field methods in multiple sites (clinical and non-clinical) were used, including participant observation and semi-structured face-to-face interviews in order to explore how the cultural context impacts notions and experiences surrounding pregnancy loss. Interviews were conducted in the hospital, at participants’ homes, or at a participant’s preferred location (e.g. café). Potential subjects were identified in the Women’s Hospital-Hamad Medical Corporation, which is the main service provider for women care in Qatar. They were consented after explaining the study to them, and being given the time to fully understand and read a printed information sheet about the project. Afterward, they were interviewed in Arabic (for about 40 min to two hours) using a semi-structured interview script. The interview script covered details about the miscarriage, notions of causation and risk, preventive measures, and feelings about the loss. It acted as prompt to allow for significant issues to emerge. The questions were framed neutrally; open-ended questions instead of leading ones were used to avoid bias [36]. The interviews were mainly conducted by two female bilingual (English and Arabic) research associates who are Biomedical Science graduates. They have five years’ collective experience in research involving women health. To ensure high quality and consistency of the data collected, the two research associates were trained by experienced anthropologists (Principal Investigator (PI) and Professor D Miller) in anthropological fieldwork methods. They were trained to the point where they demonstrated rigor associated with relevance to practice and congruence of the methodological approach. The survey questions were piloted in a sample of 5 subjects (4 women and 1 man). Piloted subjects were interviewed individually to test the appropriateness of the questions and assist the researchers to learn the skills of in-depth interviewing and the flow of conversation. This was done in English and the research associates transferred the principles into Arabic language, as they interviewed the participants.

Some women who showed willingness to meet with us again were interviewed on several occasions (from two to six times) in order to allow for detailed and nuanced data to be collected. Interviews were recorded by a digital voice recorder and then transcribed and translated (retaining key words in Arabic) soon after. The PI was involved in interviews where logistics allowed, and had frequent trips to Qatar. The research associates worked closely with the PI to ensure that high quality data is collected sensitively and effectively. The PI was also responsible of overseeing the data collection and assessing its quality via reading the interviews immediately and discussing any points for further clarification and follow up. Any key knowledge gaps informed additional interviews and fieldwork interactions. Data collection stopped at the point where additional data did not lead to any new emergent themes (i.e. until saturation was reached) [37, 38]. Where possible and appropriate, observational data was recorded in notes during the sessions. This prolonged involvement and persistent observation of participants deemed to reduce bias [39].

Interview data and other relevant documentation were analyzed though inductive thematic analysis of content by the PI and one of the research associates. The basis of the analysis was deep familiarity with the data via ongoing reading and meeting to discuss the emerging understandings. Themes were identified and sorted into categories with higher order categories emerging in line with the constant comparative method [40]. Demographics and information about marriage and kinship patterns were also collected and analyzed using simple descriptive statistics. Finally, findings were articulated with the study objectives to answer the research question. Pseudonyms were given to participants and obvious identifiers were changed to maintain anonymity.

We adhered to COREQ guidelines in our methods. For a full description of methods please refer to [32].

## Results

Among the miscarried women who were approached, forty women participated in the study; one woman was ineligible to participate as she had experienced three consecutive miscarriages in the last six months, and three women were not willing to participate. As we aimed to interview women on several occasions to build rapport and allow their detailed narrative to emerge, we managed to interview eleven women between 2 to 3 times, whereas, three women acted as key informants and were interviewed between 4 to 6 times.

Participants were all Muslims, mainly educated (95%) and employed (60%). The majority of the participants have one or more children (82.5%) and have experienced between 1 and 3 non-consecutive miscarriages (85%). Table 1 outlines primary participants’ demographics including the number of miscarriages experienced.

**Table 1:** Primary Participants’ Demographics

Characteristics	(*n* = 40)
Maternal Age (yrs)	
20–29	15 (37.5%)
30–39	21 (52.5%)
40–49	4 (10%)
Educational Level	
Illiterate	3 (7.5%)
Up to high school	12 (30%)
Higher education	25 (62.5%)
Employment	
Employed	24 (60%)
Unemployed	16 (40%)
Number of Live Births	
None	7 (17.5%)
1	6 (15%)
2–4	12 (30%)
≥ 5	15 (37.5%)
Total Number of Pregnancies	
1–2	11 (27.5%)
3–4	5 (12.5%)
≥ 5	24 (60%)
Total number of Miscarriages (non-consecutive)	
None	0 (0%)
1–3	34 (85%)
4–6	6 (15%)

Two main themes have emerged in relation to specific research questions: 1) Rhetorics of blame, self-blame, and feeling of guilt; 2) Attitudes to miscarriage.

### Rhetorics of blame, self-blame and feeling of guilt

Rhetorics of blame are activated when women lose their pregnancies [20]. When women do not fulfill their socially expected role of motherhood, they are likely to be blamed. Participants spoke about an indirect sense of blame by others. There are also voices where women felt responsible for their miscarriage and have felt guilty and ashamed as a consequence.

#### Blame

Primary participants commonly reported that others accused them of the outcome of their pregnancies, particularly in the absence of certain cause. It was suggested that they had a role in the miscarriage and that the outcome may have been different had they acted otherwise. As a result of such accusations, women reported being held accountable for the loss. Nada (20–25 years old who miscarried her first pregnancy) spoke about being blamed from people around her in an indirect way as they started to question why she had the miscarriage and what the reasons were behind its occurrence:

*When you miscarry they start asking you; “why did this happened?” I myself don’t know till now why it happened, but they think that I am hiding the reason, why do I have to hide anything?*

Similarly, the mother of a miscarriage participant (Dana; a 25–30 year-old with two children who had suffered a miscarriage and a stillbirth before experiencing a second miscarriage) confirmed by saying:

*She was telling us that her husband’s family accuses her of the miscarriage in an indirect way. Her mother in-law said; “what’s wrong with women nowadays? We used to get our babies without having any problems!”*

The findings also suggest that there are specific behaviours and activities acceptable to pregnant women [20], those who transgress these may be blamed and seen as inviting risk. Amna (age 27–32 with two children) stated that others criticized her and suggested she had performed activities they believed could have caused her miscarriage:

*Yes, in my first miscarriage, everybody blamed me. They were sure that I did something wrong. I told them that the doctor said it was not my fault but nobody believed me. They insisted that I had lifted something heavy or did something that caused the miscarriage*

The mother of another miscarriage participant (Nada; a 20–25 year-old with no children) has explicitly stated that a woman is considered culpable about her miscarriage when she contributes to the death of her baby in the womb by acting in a risky manner and not following the doctor’s recommendations:

*If she carried something heavy, in this case she is guilty, or if she took a medication that may harm her and the baby; she is guilty, or if she didn’t follow the doctor’s recommendations; she is guilty too. But if she did all what she can and followed the doctors’ recommendations and took the medications; in this case she is not guilty.*

Another miscarriage participant; Huda who is 33–38 years-old and has three children, spoke about how women are indefinitely blamed of their miscarriage:

*They always say that the woman is the only one to be blamed for the miscarriage; the hormones are from the woman; the miscarriage is from the woman…what about the man?*

Whereas the comments above are about women being held accountable for their miscarriages, Moza (*age 27–32, no children)* who experienced two miscarriages, both following IVF treatment, when asked about her thoughts about the causes of miscarriage, referred to her understanding that miscarriage is caused by being careless and not taking care of oneself when pregnant:

*Maybe the woman got exhausted by going out or doing housework or she is not taking care of herself. I know one of my relatives who was five months pregnant; she got pregnant naturally, but she didn’t seek any medical care or take any medications and she miscarried. It is all because of her carelessness*

#### Self -blame and feeling of guilt

Above, cases are outlined where others thought that women have contributed to the cause of their miscarriage, however, few women reported that they blame themselves for their unsuccessful pregnancies. When asked about the cause of her miscarriage, Noora, a 40–45 year-old participant who had miscarried recently after six successful pregnancies, stated:

*I think half of what happened was my fault. Although I had bleeding, I worked a lot at home because I don’t have a maid. People told me “you should rest and take progesterone” but I didn’t listen*

In another interview, acknowledgement of biomedical facts about miscarriage causation or certain deviant behaviours was deemed to be linked to feelings of guilt. Najah (age 30–35, no children); a participant who lost her first pregnancy, attributed her miscarriage to her work routine and blamed herself for being too active and not slowing down while being pregnant, and also for not following healthy life style:

*To be frank, I blamed myself, maybe my work rhythm was fast and I did not slow down…..you know the woman always blames herself; my lifestyle isn’t so healthy to keep that baby, I do not like vegetables and fruits, …and I don’t drink milk and these things, I blame myself for all these things*

Another participant; Ameena (age 25–30) who miscarried her first pregnancy expressed her worries with her physician about what may have caused her miscarriage, she said:

*I even asked them if I have miscarried because of walking or moving or being exhausted! If it is because of walking or climbing the stairs, then, I have to be more careful next time, rest, take care of myself and relax*

The quotations above indicate that pregnant women are expected to “take it easy” and reduce work around home and outside the home: transgressing norms about a “good pregnancy” may lead to accusations of or feelings of culpability.

Another reason for guilt feeling can be seen in Sara’s (age 27–32, 1 child) quotation. Sara who is diabetic, outspokenly announced herself guilty of the death of her baby, she blamed herself for not adhering to her medical regime knowing that she has diabetes:

*I have a feeling that I caused the death of this baby; this is a secret that I didn’t tell to anybody before! (Why?) Because they said that the high glucose level is the cause and I wasn’t taking Insulin regularly*

After having been informed by her physician that the high glucose level, which is a risk factor may have caused her miscarriage, Sara kept constantly dwelling on the idea:

*This idea kept going around in my mind since they told me that miscarriage happened because of uncontrolled diabetes.*

Whilst all participants saw miscarriage as an ordinal chosen by God [4, 16, 32], for some, this did not mean they were not feeling of guilt. There were underlying thoughts of self-blame and feelings of guilt that were triggered by other factors. Amal (37 to 42-year-old, with one child) explained that God’s will is the ultimate cause, yet she wonders if other factors may have caused her miscarriage. She considers possible causes, but dismisses them by seeing she had rested more this time as compared to her previous, successful pregnancy and so concludes that it is entirely God’ will.

*I was trying to remember if I have done anything that caused the miscarriage but eventually I say* ***Al hamdulellah [thanks God]*** *…. because while I was remembering what happened I told myself that I had much more rest in this pregnancy compared to my first one. In my first pregnancy I didn’t have a maid and my house is two floors and I used to do all the housework myself but now I have a maid. So I was trying to rationalize what happened or where I was mistaken but I didn’t find that I have done anything wrong so I said* ***al hamdulellah [thanks God]*** *; God gave him to me and then took him back!*

### Attitudes to miscarriage

In Qatar, miscarriage is seen as a normal occurrence and typically associated with empathy and support. Participants’ attitudes to miscarriage varies; while the *majority* of participants stressed that miscarriage is a relatively normal and common event, and can be shared openly [4, 32]. The following quotations reflect examples of spouse, family, acquaintances, and physician who have tried to comfort the woman who just miscarried:

*[Qatari society] is very sympathetic and considerate* (Hadeel; age 35–40 with six children)

*This is something normal and it could happen with any one* (Noora; age 38–43 with six children)

*They were continuously contacting me* via *What’s App, … and they all made me feel that what happened is normal and they have experienced it before.* ***Al hamdulellah [thanks God]*** *my mother and my husband and others were all supporting m*e (Amna; age 28–33 with two children)

*[The doctor] said this may happen in one of the pregnancies, as it is normal to fail sometimes* (Reem; age 32–37, no children)

However, this sense of miscarriage as normal was not felt by all of participants; a few women suggested that it is a taboo and should be kept private.

*There are some people who hide this and don’t talk about it! In our society they hide miscarriage, however, in other societies such as in US they sympathize with the woman. Here in Doha, it is a taboo (Aeb)! The woman who miscarries shouldn’t talk about it and shouldn’t tell anybody!* (Nada; age 20–25, no children)

Indeed, Nada’s opinion that miscarriage should be kept private reflects our previous research finding that pregnancy was often concealed [32]. Dana (age 27–32 with two children) also mentioned that concealing miscarriage is a norm in Qatar and suggested the reasons why women conceal their miscarriages:

*I think to avoid talking about it, to avoid being a topic of conversation because people feed on gossiping like this, miscarriage is usually a good topic of gossip; they gossip about ‘if it happened again’ and ‘when it happened’ and they count how many times it happened to someone, people -some people tend to collect and analyze even add some spice to it, so to avoid these people we do not want to share*

Impaired fertility can be seen as particularly problematic in Qatar, where there is an emphasis on women as reproducers [4, 16, 32]. A few number of women felt that miscarriage is resounding stigma and shame:

*They think that a woman who miscarries has something wrong; they say for sure something wrong with her, otherwise the baby would not have been lost* (Awatif; age 25–30 with one child)

*Of course they will say that she is an inadequate woman and defective and that she should be put aside* (Fareeda; age 35–40 with six children),

*Well, the same old idea is still there which is “you don’t bring kids” or “your tummy doesn’t carry babies” so once you miscarry oh my God, you have a problem and they start telling the husband “what are you going to do now?”, and they ignore that you had babies before* (Huda; age 32–37 with three children)

*yes, there is a social stigma; they say “this woman always miscarries”. For example, you may be sitting in a place with your friend and your friend points at a woman and says;" this woman has no children because they always die" or “she always gets pregnant but her babies die in the womb”* (Khadija; age 25–30 with two children)

## Discussion

The findings revealed that there was ambivalence around culpability and stigma in Qatar. This ambivalence is likely due to the interaction between the cultural and the religious factors that feature the Qatari society. Cultural and societal norms place pressure on Qatari women; the society attaches the worth of women to their ability to carry pregnancy to term and have a large number of children, [4, 16, 32]. Also, norms and values informed by Islam, a pronatalist religion, encourage reproduction and emphasize the role of a woman as reproducer and as a mother. Therefore, deviations from the expected gender role by miscarriage can be seen as a disruption in one’s reproductive life, and thereby, likely to be linked to stigma and self-blame [41]. In our study, many participants did not acknowledge feelings of guilt, however, a sense of responsibility emerges in their search for possible causes of miscarriage, suggesting elements of blame and responsibility. This, however, was not sufficient to provide conclusive evidence of the commonplace of guilt and shame, as discussed further in Kilshaw [16]. Miscarriage triggers feelings of failure in women’s responsibility and thus places them under stress and fuels feelings of guilt and self-blame. These feelings are the highest level of “internalized stigma” [42]. Stigma is defined by Goffman [43] as “a discrediting attribute that reduces someone from a whole and usual person to be a tainted, discounted one”. Women in Qatar are seen responsible for the health of their pregnancy through specific acceptable behaviours and activities [20, 32]. If they transgress or not conform to the societal beliefs about “good pregnancy” behaviours may be blamed for what is perceived as risky behaviour. The fact that there are options available and the woman have choices in them, opens opportunities for notions of risk and blame. This places a woman in a panopticon of surveillance to conform to the expected standards of behaviour.

Similar to previous research findings, blame was found to be particularly activated in the absence of certainty of cause [13, 44]. Uncertainty about causality creates opportunities for accusations or feelings of culpability [45]. When women blame themselves they rightfully hold on to their belief that they should have acted differently. This subscribes to notions of moral responsibility and indicates that acting for the wrong reasons held women culpable and morally responsible for the negative outcome. The same topic was disclosed in van der Sijpt work [9] were women blamed their lifestyle habits and attributed their miscarriages to another course of events such as illnesses and accidents.

Another factor that plays a role in feelings of guilt and shame is recognizing biological causes of miscarriage such as diabetes. Diabetes is highly prevalent in Qatar (16.7% in adult Qatari population) [46] and was widely cited by women in the discourse of miscarriage causation in Qatar [32]. Our findings revealed that those who suggested that they did not follow recommended behaviours to protect the pregnancy expressed self-blame. Both guilt and shame are self-conscious emotions involving self-blame [47] and are associated with feelings of tension, remorse, worthlessness, and powerlessness [23].

On the other hand, miscarriage is perceived as a common event. Most miscarriage women and their family members reported that the Qatari society sympathizes with them, whereas, few suggested that women who miscarry are subject to negative attitudes and societal stigma. Qatari women are comforted by their strong faith and they understand that miscarriage is ultimately caused by God and for a reason [32], which attributes to the reason it is relatively normalized and discussed openly amongst women [4, 16]. Despite this, a small number of participants did suggest that miscarriage is a taboo and should not be disclosed so that it does not become a subject of public knowledge. Women may not publicize their pregnancies until necessary, which means a subsequent loss is similarly concealed. Contrary to the Qatari context, the existing literature, which focuses on the Euro-American and Australian contexts, suggests that miscarriage is generally silenced and is handled in private [6, 8, 10].

Appreciating the sociocultural context in which miscarriage is embedded may encourage health providers to be attuned to best serve the needs of women who experience pregnancy loss. Understanding the beliefs of Qatari women and the cultural emphasis on childbearing, allows for women to receive greater individualized care and subsequently improved wellness. Attention should be paid to ensure women and those around them are given appropriate and robust information about the known causes of miscarriage, so as to deflect discourses of blame that may occur un-necessarily, and subsequently reduce avoidable harm to women who experience a miscarriage. Future research should be conducted to assess the impact of dissemination of such knowledge, in an attempt to inform public health programmes, which aim to improve women’s health literacy in the context of pregnancy and pregnancy loss, and thereby drive the efforts to reduce stigma and shame feelings that Qatari women may be experiencing un-necessarily.

## Conclusion

This paper serves as an illustration of the religious, sociocultural institutions of miscarriage aftermath. Discussion about stigma, blame, and culpability leads to inevitable ambivalence. In Qatar, the context of pronatalism and the pressures excreted on women to produce children, as well as, the role of religion in theories of miscarriage causation frames women experiences in terms of stigma, silence and openness, feeling of guilt, and culpability. A better understanding of the sociocultural context in which women interpret and give meaning to their experiences can inform strategies to improve women’s health. In particular, robust communication of causes of miscarriage would be virtuous to deflect any accusations or feelings of responsibility. It will also help in the development of culturally sensitive educational and outreach programmes. Such programmes will advance women’s health whilst reducing the burden of guilt and stigma that may be associated with pregnancy loss.

## Data Availability

The datasets generated during the current study are not publicly available due to IRB legal barriers that hindered our ability to share the data. To protect the privacy and confidentiality of the research participants, the de-identified interview scripts are available only upon request and after compliance with the policies and procedures of WCM-Q, HMC, and Qatar National Research Fund (QNRF) for data sharing. Requests can be submitted to the corresponding author on reasonable request.

## Abbreviations

COREQ: Consolidated Criteria for Reporting Qualitative Studies
IVF: In vitro Fertilization
PI: Principal Investigator
US: United States

## Footnotes

1 Pronatalism refers to a cultural emphasis on parenthood and raising children. It refers to policy and practice which suggests bearing should be the central focus of every person’s adult life [26]
